# Objective Structured Practical Examination (OSPE) as a Tool for the Formative Assessment of Practical Skills in the Subject of Physiology: A Comparative Cross-Sectional Analysis

**DOI:** 10.7759/cureus.46104

**Published:** 2023-09-28

**Authors:** Nayna R Lakum, Alpeshkumar M Maru, Chandrika G Algotar, Hardik H Makwana

**Affiliations:** 1 Department of Pathology, Gujarat Medical Education and Research Society (GMERS) Medical College, Junagadh, IND; 2 Department of Pathology, Gujarat Medical Education and Research Society (GMERS) Medical College, Morbi, IND; 3 Department of Pathology, Gujarat Medical Education and Research Society (GMERS) Medical College, Navsari, IND

**Keywords:** conventional method, reliability, validity, students, objective structured practical examination

## Abstract

Background:Practical assessments hold a critical role in evaluating medical education. However, achieving objectivity, consistency, authenticity, reliability, and practical usefulness in student evaluations can be a formidable challenge. The Objective Structured Practical Examination (OSPE) stands out as a promising technique tailored to assess performance in a realistic educational setting. OSPE offers a unique approach to aligning assessment methods with the educational objectives of a given activity, making it possible to comprehensively gauge the attainment of pedagogical goals.

Objective: This study aimed to overcome the limitations associated with traditional practical tests and explore the potential advantages of OSPE in improving the objectivity, consistency, authenticity, and reliability of student evaluations in the context of medical education. Through a comparative analysis, this research endeavors to illuminate the practical applicability of OSPE. The primary goal of this research was to introduce and assess the feasibility of employing the OSPE as a formative assessment tool for appraising the practical capabilities of Physiology students.

Methodology: Fifty students from 1st year MBBS were included in this study after their written consent. They were divided into two groups of 25 students each; two practical procedures, (a) hemoglobin estimation, and (b) performing blood group. Students were assessed at two different sessions. Students of each group assessed by the conventional method in the first session were assessed by OSPE in the second session of the same practical and vice versa. At the completion of the assessment process, both students and teachers were asked to rate the various assessment techniques on a Likert scale. Student test results and instructor and student opinions were statistically examined using the paired t-test. A significance level of 0.05 was used.

Results: When evaluated using the OSPE method, students obtained significantly higher mean marks (12.58±2.74) compared to the conventional assessment method (8.44±2.13). A paired t-test confirmed the statistical significance of the improvement in student performance with OSPE (p<0.0001). Student feedback indicated strong agreement (92%) that OSPE encourages greater focus on practical examinations and is an effective assessment and learning method. Teachers expressed unanimous agreement that OSPE is a more comprehensive evaluation tool (100%) and better at highlighting student strengths and weaknesses (75%). The majority of teachers (75%) believed that OSPE should be incorporated into future examinations.

Conclusion:* *The study demonstrates that OSPE significantly enhances student performance and is well-received by both students and teachers as a more effective and comprehensive assessment method.

## Introduction

The purpose of any evaluation procedure is to determine how well pupils have learned their intended content [1,2]. This means that every kind of assessment must have a clear connection to the aims of the course. Current methods of evaluating students' performance in physiology labs are obsolete since they focus on knowledge that is unnecessary for the development of a competent physician. The practical test is a crucial part of the medical education assessment process. However, if requirements like impartiality, consistency, validity, dependability, and practicability are to be satisfied, student assessment becomes a difficult task [1]. Presently, at most medical institutions in India, practical exercises in physiology are done and assessed in the traditional manner, i.e., a student is given an experiment to execute, a viva is held after the practical exercise is completed, and the candidate is then evaluated.

There are a number of methods for making this more uniform, but the Objective Structured Practical Examination (OSPE) is often used. Harden and Gleeson's Objective Structured Clinical Examination (OSCE) is the inspiration for this approach [3]. The OSPE seems to be a valid tool with a high capacity for distinguishing various types of pupils. In these ways, it excels above the standard practical test. In addition, it may be organized in a manner that allows for the assessment of all laboratory teaching goals while giving due consideration to all relevant factors [4].

The human physiology traditional clinical examination (TCE) consists of a bedside viva and an evaluation of the candidate's overall performance rather than a test of their specific clinical abilities after they have completed a predetermined clinical procedure [5]. According to Miller's hierarchy of skills, TCE prioritizes the "knows" and "knows how" levels [6]. OSPE, on the other hand, is concerned with the apex of the pyramid.

Traditional approaches to evaluating competence are not only more open to bias than objective measures, but they also exclude the assessor from seeing the candidate in action. In addition, there may be restrictions on what is really covered. Sensitization towards a new evaluation system of the OSPE is required, as is a more objective and organized assessment approach, feedback from the students, and feedback to the students to recognize their deficiencies and enhance their clinical abilities.

So, many have tried to come up with new methods to get around such problems. The OSPE is one such approach. All of an activity's pedagogical goals may be gauged thanks to the way the assessment was set up in this case. This research aimed to spread OSPE across the physiology department as a novel evaluation tool.

## Materials and methods

The research design was a comparative cross-sectional analysis. It took place in Junagadh, India, in the Physiology Laboratory of the Gujarat Medical Education and Research Society (GMERS) Medical College. Between May and July of 2016, the research was conducted. Fifty MBBS students in their first year served as the study population. Students were included in the research if they agreed to take part in it, whereas those who said no were left out.

Written informed consent from the students was obtained before the research, and approval was granted by the institution's ethics committee (IRB number: IEC/GMERS/2016/11). Then, a month before the OSPE assessment, a brief lecture and role play were used to familiarize staff and students with the assessment technique. Fifty first-year medical school students agreed to take part in the research. The time just before the last formative evaluation was chosen for analysis. The effectiveness of two real-world methods was evaluated. Hemoglobin count and blood type determination fell into that category. A subject matter expert was consulted in order to develop an outline of the course material and a systematic checklist for both observed and unobserved stations in accordance with Bloom's taxonomy. Group A consisted of 25 pupils, while Group B consisted of the remaining 25 students. In all, four assessors were assigned to the task, split evenly between Groups I and II. The test was broken up into two sessions (Table 1).

**Table 1:** Examination conducted CPE = Conventional Practical Examination OSPE = Objective Structural Practical Examination

Day	Teachers Group I	Teachers Group II
Day 1 (Session 1)	Conducted CPE of Group – A (HB estimation)	Conducted OSPE of Group – B (Blood group testing)
Day 2 (Session 2)	Conducted CPE of Group – B (blood group testing)	Conducted OSPE of Group – A (HB estimation)

At the end of both sessions, Group A students performed hemoglobin estimation and were assessed by both methods. Group B students performed blood group and were assessed by both methods. Each student was exposed to a different set of examiners in both exams to reduce the chances of bias.

In the traditional assessment method, students performed practical exercises followed by viva. In OSPE, students were given comprehensive pre-exam training. There were many stations in OSPE, and participants had limited amounts of time to finish each one. The stations focused on observation, procedure, and brief questions to test not just psychomotor skills but also higher-order thinking. Each checkpoint was planned in tandem with the inspection checklist.

After the test, the scores from the classic practical were compared to those from the OSPE. Using a pre-validated survey, we also collected and evaluated data on how students and teachers felt about OSPE. All of the survey questions were of the 5-point Likert kind. In ascending sequence, these points represent how much you agree with the statement in question.

The data was examined using the paired t-test, with the results (marks) reported as mean and standard deviation. The input was given in the form of percentages based on a 5-point Likert scale. A significance level of 0.05 was used. The statistical analysis was performed using Statistical Package for Social Sciences (SPSS), version 16 (SPSS Inc, Chicago, IL).

## Results

Fifty students taking the midterm test were included in the current research, and their performance was evaluated by four instructors. Student performance as measured by several evaluative modalities. During the examination, the mean marks obtained by students with assessment by the conventional method were 8.44±2.13 whereas it was 12.58±2.74 with the OSPE method (Table 2).

**Table 2:** Comparative marks obtained by students assessed by different assessment methods (maximum marks - 20) *paired t-test OSPE = Objective Structural Practical Examination CPE   = Conventional Practical Examination

Method	N	Minimum Marks Obtained	Maximum Marks Obtained	Mean	SD	T-value	p-value*
Conventional	50	4	13	8.44	2.13006	11.21	0.0001
OSPE	50	5	18	12.58	2.74115		

The results of the current research demonstrated that when compared to the traditional technique of evaluation, pupils received considerably better grades when examined using OSPE. Students' improved performance while using the OSPE approach was statistically confirmed by a paired t-test, with a p-value of 0.0001, much below the significance level of 0.05 (this holds true at the .05 level).

Students' opinions on the relative merits of the two testing procedures, as expressed by their ratings on a 5-point Likert scale, are summarized in Table 3.

**Table 3:** OSPE feedback form and response from students OSPE = Objective Structural Practical Examination

No.	Parameter	Strongly Agree Number (%)	Agree Number (%)	Neutral Number (%)	Disagree Number (%)	Strongly Disagree Number (%)
1	OSPE encourages us to pay more attention to practical examination	19 (38%)	27 (54%)	0 (0%)	2 (4%)	2 (4%)
2	OSPE is a good form of examination and learning process	27 (54%)	22 (44%)	1 (2%)	0 (0%)	0 (0%)
3	OSPE covers the relevant and important topics and is consistent with the learning objectives of the syllabus	16 (32%)	25 (50%)	8 (16%)	1 (2%)	0 (0%)
4	Checklists provide a fair and unbiased system of marking	25 (50%)	17 (34%)	4 (8%)	3 (6%)	1 (2%)
5	OSPE reduces the element of luck in the examination	27 (54%)	15 (30%)	5 (10%)	3 (6%)	0 (0%)
6	OSPE is easier to pass the exam compared with conventional method	28 (56%)	20 (40%)	2 (4%)	0 (0%)	0 (0%)
7	OSPE is more stressful compared with conventional method	4 (8%)	12 (24%)	6 (12%)	5 (10%)	23 (46%)
8	Attitude of teacher during OSPE was better compared with conventional method	19 (38%)	24 (48%)	6 (12%)	1 (2%)	0 (0%)
9	OSPE should be used as a method of assessment in future examinations	21 (42%)	23 (46%)	4 (8%)	1 (2%)	1 (2%)
10	OSPE is a better way to assess the different aspects of knowledge	19 (38%)	21 (42%)	5 (10%)	1 (2%)	4 (8%)

The following information is gleaned from student feedback surveys. Ninety-two percent of students feel that OSPE motivates them to focus more on practical tests, while 8% disagree. Almost all of the students (98%) agree that OSPE is an effective method of testing and education. Eighty-two percent of students feel that OSPE adequately covers the material and aligns with course goals, while 2% strongly disagree. More than half of students (56%) say that compared to the traditional technique, OSPE is more demanding. During OSPE, 86% of students said they felt their teacher's demeanor improved. The majority of students (80%) believe that OSPE is a more accurate reflection of their knowledge in all areas. The vast majority of students (84%) believe that OSPE makes exams more fair. The vast majority of students (88%) feel that OSPE testing should be included in the next exams.

Teachers' input on a 5-point scale about a comparison of the two evaluation methods The details of the Likert scale are shown in Table 4.

**Table 4:** OSPE feedback form and response from teachers OSPE = Objective Structural Practical Examination

Questions	Strongly Agree Number (%)	Agree Number (%)	Neutral Number (%)	Disagree Number (%)	Strongly Disagree Number (%)
OSPE covers a wider range of knowledge as compared with conventional examination	3 (75%)	1 (25%)	0	0	0
OSPE compels the student to learn different procedures in detail	4 (100%)	0	0	0	0
OSPE specifically highlights the weak and strong parts of subject of student	1 (25%)	2 (50%)	1 (25%)	0	0
OSPE is more stressful compared with conventional method	0	3 (75%)	1 (25%)	0	0
OSPE is more exhausting compared with conventional method	0	3 (75%)	1 (25%)	0	0
OSPE is a better way to assess the different domains of knowledge of student	3 (75%)	1 (25%)	0	0	0
Checklists in OSPE provides a fair system of marking	1 (25%)	2 (50%)	1 (25%)	0	0
The factor of variability of examiner can be removed in better way by OSPE	3 (75%)	0	1 (25%)	0	0
OSPE should be used as method of assessment in future examinations	2 (50%)	1 (25%)	1 (25%)	0	0

One hundred percent of educators in this sample think that OSPE is more comprehensive than traditional testing methods. One hundred percent of educators feel that OSPE forces their students to master certain practices. Seventy-five percent of educators believe OSPE is a more stressful evaluation approach since it calls attention to both students' subject-area strengths and weaknesses. One hundred percent of educators think OSPE is a superior tool to evaluate students' proficiency across a variety of subject areas. Seventy-five percent of educators believe OSPE eliminates examiner variability in testing. Seventy-five percent of educators believe that OSPE should be included in the next exams.

## Discussion

OSPE is now administered as either a formative or summative test in a small subset of India's medical schools [6]. Although both theoretical and practical examinations are used to evaluate pathology students, we have a clear advantage in administering theoretical examinations and doing them with more consistency [7]. This research set out to compare two commonly used approaches to grading students' performance on two lab exercises administered as part of a physiology course's practical test. Blood group testing and hemoglobin measurement were the two hands-on labs students participated in for their OSPE and conventional practical examination (CPE) grades. Students and educators who took part in the study provided their feedback. The study's major objective was to implement the OSPE evaluation strategy at the university. Teacher and student familiarity with the OSPE evaluation approach is aided by the comments received. This will serve as the foundation for further development of the OSPE as an evaluation tool. Students using OSPE in this research received much better grades than those receiving traditional evaluations. Rehana et al. found similar outcomes in their research [8]. This might be because the OSPE technique is more objective than the traditional approach, which relies heavily on the assessor's personal opinion. Reduced examiner bias may be attributed in part to the objective nature of the evaluation process.

Learners are given an opportunity to share their thoughts and opinions via a process known as feedback [9]. A variety of changes may be made to enhance the quality of instruction and evaluation based on student feedback [10]. According to the results of this study's analysis of student comments, OSPE is seen as less stressful and tiring than traditional PE. The results agree with those of a research study by Rehana et al. in which objective structured practical examinations (OSPE) and viva voce are used to evaluate students' competence in the physiology laboratory [8]. Analysis of student answers also revealed that they found the procedure to be simple, standardized, fair, stress-free, and objective, and they advocated for its continuance as an evaluation tool for practical examinations [8]. During OSPE, educators had a more positive outlook on assessment, too. The vast majority of students are in favor of using OSPE in upcoming exams since they believe it is a valid and reliable means of evaluation. The vast majority of students think OSPE is an accurate reflection of their knowledge and that it has an effect on how they study. A similar pattern of results was found in a research study comparing OSCE and conventional examination (CE) as a formative assessment method in pediatrics in semester tests for final-year MBBS students by Mondal et al. [11]. Almost identical to the current survey, the previous one found that 73.8% of students thought that objective structured clinical examination was a superior formative evaluation technique than conventional test, whereas 9.5% of students preferred traditional examination [11]. Based on these findings, it can be concluded that OSPE is generally regarded as a preferred form of evaluation by students.

Teachers' opinions are generally in agreement that OSPE is preferable because it tests students on a broader range of material than a traditional exam, requires them to learn new procedures in depth, and draws attention to both students' areas of strength and weakness in a given subject. Most educators agree that OSPE is a more consistent way to evaluate students in the classroom, hence it should be utilized in standardized tests going forward. The vast majority of educators, however, see OSPE as a more taxing and nerve-wracking way to evaluate students. This might be because of the time and effort required to create customized checklists for evaluating different types of practical activities.

In order to provide an effective and accurate assessment of student performance in practical situations, a modernized testing system is required. Experiential learning is an important part of a doctor's professional development and continues throughout their careers [12]. The primary goal of medical school is to provide students with the knowledge necessary to comprehend the underlying physiological changes that manifest as illness [13]. The basic goal of medical education is to produce professionals who are up to the task of treating patients [14]. OSPE assisted students who performed below or above average on tests of cognitive, psychomotor, and attitude skills, suggesting that it is a reliable strategy with a strong capacity to distinguish between various groups of pupils.

The study limitations include limited resources, lack of time, and a smaller sample size. Long-term effects on student learning outcomes or retention were not explored. A follow-up study could provide insights into the sustainability of the observed improvements.

## Conclusions

In light of our dedicated efforts, our academic institution now possesses a valuable asset in the realm of physiology education innovative evaluation method known as Objective Structured Practical Examination (OSPE). Demonstrably superior to conventional assessment methods, OSPE should become the standard against which future examinations are measured. Its integration into upcoming assessments not only aligns with the evolving requirements of medical education but also meets the desires of both educators and students for more effective and comprehensive evaluation tools. OSPE offers the prospect of cultivating a deeper understanding of practical skills and subject mastery, equipping students with the competencies essential for success in their medical careers.

As we progress, it becomes imperative to continue researching and evaluating the efficacy of OSPE, its adaptability to various medical disciplines, and its long-term impact on student learning outcomes. This study marks the initiation of a transformative journey in medical education, where innovation and evidence-based assessment practices chart a course toward the development of more proficient and capable medical professionals for the future.
